# Epidemiological, Molecular, and Clinical Features of Norovirus Infections among Pediatric Patients in Qatar

**DOI:** 10.3390/v11050400

**Published:** 2019-04-29

**Authors:** Shilu Mathew, Khalid Alansari, Maria K. Smatti, Hassan Zaraket, Asmaa A. Al Thani, Hadi M. Yassine

**Affiliations:** 1 Biomedical Research Center, Qatar University, Doha 2713, Qatar; shilu.mathew@qu.edu.qa (S.M.); msmatti@qu.edu.qa (M.K.S.); aaja@qu.edu.qa (A.A.A.T.); 2 Pediatric Emergency Center, Hamad Medical Corporation, Doha 3050, Qatar; dkmaa@hotmail.com; 3 Department of Experimental Pathology, Microbiology, and Immunology, Faculty of Medicine, American University of Beirut, Beirut 1107 2020, Lebanon; hz34@aub.edu.lb

**Keywords:** norovirus, genotyping, age-specific, severity

## Abstract

Background: Norovirus (NoV) is recognized as the second most important etiological agent leading to acute gastroenteritis globally. In order to determine the burden and characteristics of NoV infections in children in Qatar, profiling of circulating genotypes and their correlation with demographics and clinical manifestations were evaluated. Methods: A total of 177 NoV-positive fecal samples were collected from children suffering from acute gastroenteritis (AGE) during two-year period between June 2016 and June 2018. The age of the subjects ranged between 3 months and 12 years (median of 15 months). Genotyping was performed by amplifying and sequencing parts of viral VP1 and RNA-dependent RNA polymerase (RdRp) regions. Phylogenetic analysis and evolutionary relationships were performed using MEGA7.0. Fisher’s exact test was used to run statistical analysis for the clinical and demographical characteristics of circulating strains. Results: Overall, NoV infections were relatively higher in males than females with a ratio of 1.3:1 (*p* = 0.0073). Most of the NoV infections were reported in children between 1 and 3 years old (49.7%), followed by those <1 and >3 years of age (41.2% and 9.1%, respectively). NoV infections occurred throughout the year, with a noticeable increase in summer (36.6%) and drop in winter (25.4%). Nearly all (98.8%) NoV-infected children were positive for genogroup II (GII) compared to only two samples (1.2%) being positive for genogroup I (GI): GI.3 and GI.4. NoV genotype GII.4 (62.2%), GII.2 (15.8%), and GII.3 (13.5%) were predominant in our study. The detected strains shared >98% sequence homology with emerging recombinant strain of GII.P16-GII.4/RUS/Novosibirsk/2017 (MG892929), GII.P16-GII.4 Sydney/2012 (KY887601), GII.4 Sydney/2012, recombinant GII.P4 New Orleans /2009/GII.4 Sydney 2012 (MG585810.1), and the emerging strain GII.P16-GII.2 CHN/2017 (MH321823). Severe clinical illness (vesikari score >10) was reported in children infected with genotypes sharing homology with the above emerging strains. While GII.4 was reported in all age groups, NoV GII.3 infections were higher in children <1 year of age. Both genogroups (GII.4 and GII.3) in addition to GII.2 reported higher incidence in Qatari subjects compared to other nationalities (*p* = 0.034). Conclusion: This is the first report about NoV molecular epidemiology in Qatar. The most detected NoV strain was genogroup GII, which is the dominant genotype in the Middle East region. Further, we report GII.4, GII.2, and GII.3 as the most predominant NoV genotypes in our study. Moreover, disease severity scores were higher among children genotyped with genogroup GI (GI.4) and genogroup GII (GII.4, GII.2, GII.3, GII.6, and GII.7).

## 1. Introduction

Noroviruses (NoVs) belong to the family Caliciviridae and are the leading cause of acute gastroenteritis worldwide [1]. Transmission occurs through the fecal–oral route and is facilitated by the virus environmental persistence and low infectious dose [2]. Following an average incubation period of 24 to 48 h, signs of NoV infection start to appear, which include diarrhea, projectile vomiting, fever, dehydration, and abdominal cramps.

Until recently, human NoVs were hard to grow in vitro. Recent reports have shown that NoVs could be cultured in stem cell-derived enteroids [3] and in human B cells [4]. Moreover, a replication model in zebrafish larvae was also recently developed [5]. Nonetheless, simpler cell culture models are still not available. Accordingly, investigations of the etiologic role of NoVs in acute gastroenteritis (AGE) are majorly based on the use of sensitive molecular techniques such as reverse transcriptase-polymerized chain reaction (RT-PCR) [6]. Further, the widespread use of molecular techniques has provided better knowledge on the diversity of NoV genotypes and strains circulating worldwide [2,6].

NoV has three open reading frames (ORF): ORF1 encodes the viral RNA-dependent RNA polymerase (RdRp), ORF2 encodes the viral protein (VP1) and ORF3 encodes the minor capsid protein (VP2) [7]. The VP1 consists of two main domains: the inner shell and the protruding arm, which is further divided into the P1 and P2 subdomains [8]. The surface-exposed P2 subdomain is hypervariable and it contains the main antigenic and histo-blood group antigen (HBGA) binding sites. These factors play an integral role in determining susceptibility to NoV infection [9]. Since the mid-1990s, NoV genotyping has been done based on RNA-dependent RNA polymerase (RdRp) and major viral capsid protein (VP1) gene sequencing [10]. Genotypes are typically assigned with a threshold similarity greater than 80% among viruses of the same genogroup [11]. However, the high rate of mutation and recombination in NoV-VP1 region poses a particular challenge for molecular analysis [12,13,14]. Based on the amino acid (AA) sequence of VP1, NoV is characterized genetically into genotypes I to V, which are further divided into more than thirty genotypes [15]. In humans, genogroup II has the highest circulating frequency worldwide, with genotype GII.4 being generally predominant and responsible for the majority of annual outbreaks [6]. New strains of GII.4 are reported to emerge every 2 to 3 years replacing the circulating pandemic strains, compared to the less frequently circulating GII.3, GII.6 and GII.17 strains [16,17,18,19]. Furthermore, the GII.4 lineage had on average a 1.7-fold higher rate of evolution within the capsid sequence compared to other genogroups, supporting the theory that it is undergoing antigenic drift at a faster rate [20]. GII.3 and GII.6 are also reported as predominant genotype (ORF 2) after GII.4, respectively [21,22]. NoV infection, mainly with GII.3 and GII.4, has also been reported in nonsecretors phenotype due to the absence of HBGAs expression involved in the attachment of NoV to intestinal cells [4].

While the incidence and the molecular variability of NoV-associated AGE are well established in many parts of the world, limited data exist on the disease burden and the circulating genotypes in the Middle East and North Africa (MENA) region. So far, 25 studies reported the circulating genotype associated with NoV gastroenteritis, in which only three studies were conducted on NoV among all age groups in the MENA region. A recent review suggested that children less than 5 years old in the MENA region bear the highest burden due to NoV infection [20]. NoV GII.4 was found to be the predominant genotype among all age groups in the MENA region. According to the available data, NoV seems to peak in the winter season in ~40% of the MENA countries (Egypt, Morocco, Tunisia, and Turkey), while it still circulates during the fall and summer in 25% of the countries (Egypt, Libya, Morocco, Tunisia, and Turkey) [23]. This differential seasonality is affected by variations in environmental conditions, temperature cycles, as well as winds and humidity rain patterns [24].

Qatar is a multinational country where more than 85% of its population are expats arriving from more than 60 countries around the globe [25]. This may present a threat for introducing various kinds of pathogens into the country, and hence it is necessary to run active surveillance programs to understand the epidemiology of these pathogens and prevent their spread locally. This becomes more important considering Qatar is preparing for World Cup 2022. Although screening for AGE agents is regularly performed in Qatar hospitals, little is known about their molecular characteristics and clinical outcomes. A previous prospective study from Qatar in 2013 [26] identified NoV as the leading agent (28.5%) of AGE among adult and young patients admitted to emergency departments. However, the study was limited to one year and did not investigate the molecular and epidemographic characteristics of identified agents. Here, we report the incidence of NoV and its genotype distribution among children in Qatar during a two-year period. We also analyzed the correlation between circulating genotypes and clinical manifestations as well as the demographics of the studied population.

## 2. Materials and Methods

### 2.1. Sample Collection, Characteristic, and Follow-Up

This is a two-year hospital-based study on NoV-associated AGE conducted between June 2016 and June 2018. Stool samples were collected from children admitted to Pediatric Emergency Center (PEC) of Hamad Medical Corporation (HMC; the major hospital in Qatar), with signs of AGE (frequent diarrhea (>2) and vomiting in last 24 h) and tested positive for NoV infections. IRB approval for the study was obtained from Hamad Medical Corporation (#16173/16) and a written informed consent was obtained from the parents of the children to use their samples in the study. In addition to the samples, we also collected demographic (age, gender, and nationality) and clinical data of the enrolled children. Clinical data sheet included information about duration/frequency of both diarrhea and vomiting, fever, date of symptoms onset, admission and discharge dates, antibiotics and other treatments, rotavirus vaccination, neurological symptoms, degree of dehydration, and underlying illnesses. All NoV-infected patients (*n* = 177) were followed up to seven days and clinical manifestations were reported.

### 2.2. Processing and Extraction of Viral RNA

Samples from patients with AGE were initially screened with Film Array Gastrointestinal (GI) Panel kit (BIOFIRE®, Cambridge, USA) at HMC and samples positive for NoV were then transferred to Qatar University Biomedical Research Center for further molecular characterization. Half a gram of stool sample was suspended in 4 mL of sterile 10% phosphate-buffered saline (PBS) in water. The fecal suspension was then vortexed and centrifuged for 20 min at 4000× *g*. The supernatant was collected and transferred into a new 15 mL tube before being centrifuged again at 1500× *g* for 10 min, following which, 240 µL of the supernatant was used for viral RNA extraction using QIAamp Viral RNA extraction kit (QIAGEN, Hilden, Germany) according to manufacturer’s instructions. Concentrations of extracted viral RNA were measured by Infinite 200 PRO NanoQuant Plate™ - Tecan (NanoQuant, Woburn MA, USA). RT-PCR amplification comprising partial RdRp and VP1 genes of the virus was generated using primers G1SKF (FP-CTGCCCGAATTYGTAAATGA) and G1SKR (RP- CCAACCCARCCATTRTACA) for the GI genogroup; and COG2F (FP- CARGARBCNATGTTYAGRTGGATGAG) and G2SKR (CCRCCNGCATRHCCRTTRTACAT) for the GII genogroup [27]. Viral RNA that failed to generate a PCR product was subjected to a separate round of PCR using G1SKF2 (ATGATGATGGCGTCTAAGGACGC) and G1SKR2 (CCAACCCARCCATTRTACATYTG) for the GI genogroup: and G2SKR (FP- CNTGGGAGGGCGATCGCAA) and G2SKR (RP-CCRCCNGCATRHCCRTTRTACAT) for the GII genogroup [27,28]. Briefly, 2 µL of purified viral RNA was used as a template in a 20 µL total reaction volume using Qiagen One-step RT-PCR Kit. An RT step was carried out at 50 °C for 30 min, followed by initial PCR activation step at 95 °C for 10 min; denaturation at 95 °C for 30 s, annealing at 42 °C for 30 s, and extension at 72 °C for 30 s (35 cycles); and final extension at 72 °C for 10 min. The amplified products were detected by gel electrophoresis on a 1.5% agarose gel, containing 4 µL of ethidium bromide, using the BioRad gel documentation system (Bio-Rad, Hercules CA, USA). The expected PCR product size for genogroup GI-, GII-, and GII- negative samples were 330 bp, 350 bp, and 387 bp, respectively. PCR products were stored at −20 °C until further analysis.

### 2.3. NoV Sequencing and Genotyping

Purification of the PCR products was performed using manual PCR clean up steps as previously described [29]. Briefly, PCR products were mixed with 27.5% of 100% ethanol, 1.5 µL of sodium acetate (3M, pH: 5.2), and 1.5 µL of EDTA (0.5 M, pH: 8), mixed by vortexing and centrifuged at maximum speed for 30 min. The supernatant was discarded and first PCR purification was done by adding 100 µL of 70% ethanol to each sample, vortex and centrifuged at maximum speed for 10 min. The supernatant was discarded and samples were air dried at room temperature overnight before resuspending with 15 µL of nuclease-free water. Nucleotide sequencing of the first purified product was performed at MCLAB (South San Francisco, CA, USA) using the PCR primers. The obtained sequences were utilized to determine the genotype using the web based Norovirus Genotyping Tool Version 1.0 software [30]. Multiple sequence alignments were done using CLUSTALW 2 (https://www.genome.jp/tools-bin/clustalw) and phylogeny trees were constructed with Molecular Evolutionary Genetics Analysis Version 7.0 (MEGA 7) software using the neighbor-joining approach validated by replicating with 1000 bootstraps as previously reported [31]. The reference strains for GI and GII were obtained from the GenBank and Noronet database to demonstrate the relationships between NoV genotypes from the outbreaks and globally circulating strains.

### 2.4. Vesikari Score System for Severity of NoV Infection

We evaluated the disease severity of NoV AGE infected children by applying the Vesikari score system [32] according to clinical manifestations: total score < 7 considered mild; 7–≤10 considered moderate; and >10 (≤20) considered severe. The scoring system considered general AGE symptoms: duration of diarrhea and vomiting episodes, temperature, and dehydration (mild dehydration: treat at home; moderate dehydration: treat using oral rehydration salts, 1–5% loss body weight, and severe dehydration: treat using IV therapy, ≥6% loss body weight) [32].

### 2.5. Statistical Analysis

All statistical data analysis was done using GraphPad (Prism version 5.04) (IBM, Armonk, NY, USA). Fisher’s exact test was used to determine significant differences for comparisons of general categorical variables. *p*-value < 0.05 were considered to be statistically significant.

## 3. Results

### 3.1. Demography and Clinical Outcomes of the Study Subjects

**Figure 1:**
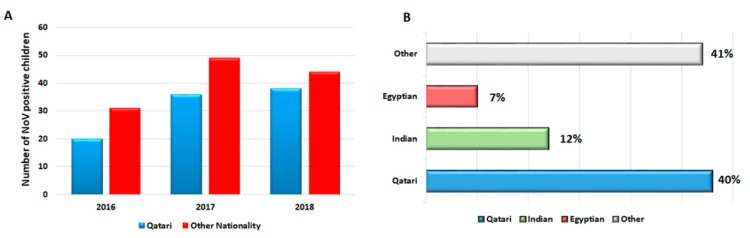
Overall NoV incidence in children admitted with AGE to emergency center. (**A**) Number of NoV-positive infections among Qataris (blue color) and other nationalities (red color) represented as bar chart. (**B**) The bar chart illustrates the top three nationalities with NoV infections: Qataris (blue color), Indians (green color), Egyptians (marron color), and other nationalities (gray color). The percentage of total cases is denoted in X-axis (left) and nationalities in Y-axis (right).

Between June 2016 and June 2018, 600 children were admitted to the emergency ward with AGE symptoms, of which, 177 (29.5%) were positive for NoV infection. Table 1 denotes the demographic characteristics of the study population. The proportion of males to females infected with NoV was 1.3:1. Moreover, the highest rate of NoV infections was reported in children between 1–3 years of age (49.7%), followed by < 1 year (41.2%) and > 3 years (9.1%) (Table 1). As expected, the majority of NoV-infected children had symptoms of diarrhea (99%), dehydration (98%), vomiting (93.3%), and fever (39.5%). Only 4.5% of infected children showed severe dehydration, whereas approximately 43% and 50.2% showed moderate to mild dehydration, respectively (Table 2). The duration of hospitalization of NoV-infected children ranged from two to four days (average: 3 days, standard deviation (SD): 1.56). According to the Vesikari score, 27 (21.2%) children reported mild symptoms (score  <  7), 76 (60%) with moderate (7 ≤ scores ≤ 10), and 24 (18.8%) with severe (score ≥ 10) symptoms. During the follow-up period for about one to seven days, only 24 (13.6%) children were prescribed for treatment (ceftriaxone, paracetamol, cefexime, electrolyte replacement solution, IVF, salbutamol, domperidone, NaCl, and ibuprofen). From these, 22 (91.7%) children were fully recovered, while only two (8.3%) did not recover completely by the end of the follow-up period. Moreover, both unrecovered children had one of their siblings affected with AGE infection. As for children who did not receive treatment, 84.3% were fully recovered within 6 days, while 15.7% did not recover completely during the follow-up period (7 days) (Table 2). Out of the total number of NoV-positive cases (177/600), 31 (17.5%) were detected during second half of 2016, 78 (44%) in 2017, and 68 (38.4%) during the first half of 2018 (Figure 1A). The NoV incidence rate was lesser among Qatari subjects compared to other nationalities. The highest three rates of NoV infections were reported in Qataris (40%), Indians (12%), and Egyptians (7%), respectively (*p* = 0.04) (Figure 1B).

### 3.2. Seasonality of NoV Infections

**Figure 2:**
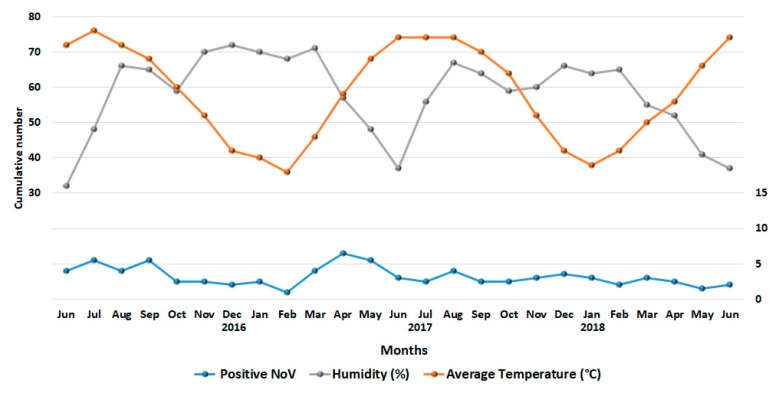
Stacked line chart denoting cumulative number of NoV-positive cases detected with temperature and humidity in Qatar during the study period (June 2016 to June 2018). X-axis: month; y-axis: cumulative number. Number of positive cases is represented in blue color, temperature (°C) in orange color and humidity (%) in gray color between June 2016 and June 2018 in Qatar.

Stacked line chart denoting cumulative NoV infections during the study period, along with records of temperature and humidity are shown in Figure 2. Although the number of NoV infection occurred throughout the year, a drop in NoV infections was recorded between October and December (the winter season, average temperature: 22 °C and average humidity: 66%) comprising 25.4% (45/177) of the overall infections. Moreover, a rise in NoV cases was noticed in warmer months between May and August (average temperature: 37 °C, average humidity: 48%). The NoV reported cases among children during these four months represented 36.6% (64/177) of the total number. Such seasonal patterns of NoV infection were the same during the study period from June 2016 and June 2018.

### 3.3. Genetic Variability of NoV Infections

**Figure 3:**
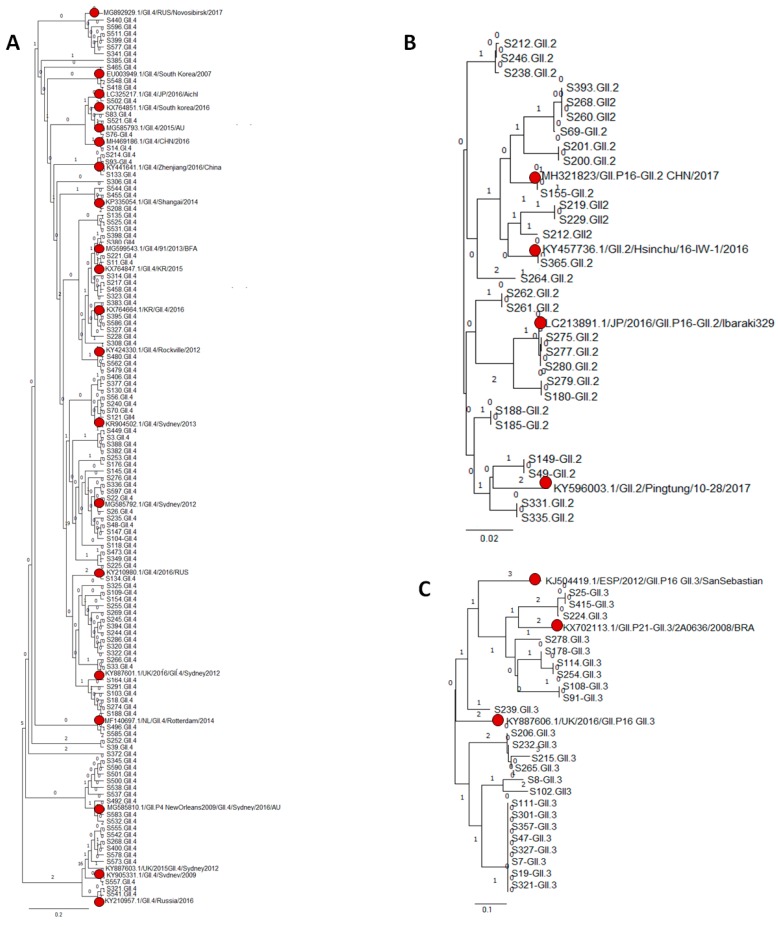
Phylogenetic tree construct based on partial sequences of the capsid and RdRp gene of NVs. (A) Phylogeny analysis of NoV GII.4 circulating strain in children in Qatar. (B) Phylogeny analysis of NoV GII.2 circulating strains (C) Phylogeny analysis of NoV GII.3 circulating strains. The evolutionary tree was constructed by using Neighbor-Joining method with 1000 bootstrap with MEGA7 [32]. The evolutionary distances were computed using the Poisson correction method and are in the units of the number of amino acid substitutions per site. Reference strains are represented in marron color spheres.

We were able to sequence and subsequently genotype all 177 NoV-positive samples included in our study. In order to analyze the circulating genotypes among NoV-infected children, we inferred the phylogenetic analysis along with the reference sequences from GenBank and Noronet. Two samples were documented as GI based on their clustering in the phylogenetic analysis; of which, one was designated as GI.3 and the other as GI.4. Identified G1.3 strain was closely related to the South Africa/2016 (MF182349.1) strain (95% nucleotide identity) [33], while GI.4 shared sequence similarity (98% nucleotide identity) to the Venda/2015 (KY012280.1) strain [34] (Supplementary Figure S1). On the other hand, nine different NoV GII genotypes were identified among the infected children, of which 62.2% were identified as GII.4. GII.4 strains clustered closely with emerging recombinant strain GII.P16-GII.4/RUS/Novosibirsk/2017 (MG892929), followed by GII.P4 New Orleans/2009/GII.4 Sydney/2012 (MG585810.1), GII.P4-GII.4/Rotterdam/2014 (MF140697.1), GII-4 /South Korea/2016 (EU003949.1), and GII.P16-GII.4 Sydney/2012 (KY887601), with 98% nucleotide identity (Figure 3A).

Likewise, based on the phylogeny analysis, GII.2 strains were closely related GII.2/Pingtung/10-28/2017 (KY596003.1) and emerging strain GII.P16-GII.2 CHN/2017 (MH321823)**,** with 98% nucleotide identity (Figure 3B). GII.3 was the third most predominant genotype in our study and strains from this genotype were closely related (98% nucleotide identity) to the viral strains from Brazil/2008 (KY702113.1), United Kingdom/2016 (KY887606.1), and Spain/2012 (KJ504419.1) (Figure 3C). The number of amino acid substitution per sequenced site in NoV GII.4, GII.3 and GII.3 identified were 0.05, 0.02, and 0.1, respectively. However, all of the RdRp sequenced in our study for GII.3 and GII.2 are of the GII.P16 genotype.

### 3.4. NoV Genotypes and Age of Children

We identified the association of specific NoV genotypes and the age of infected children. GII.4 was detected at higher rates among children from 1 to 3 years of age (55%) compared to other age groups (Table 3). Likewise, GII.3 was detected in high level among children of age group 1 to 3 years representing 67.9% of the infected children **(**Table 3). On the other hand, most of the detected GII 2 occurred in children younger than one year of age (75%). Furthermore, NoV uncommon genotypes GII.6 and GII.17 were detected among all three age groups with no significant differences. GII.7 was detected in one child in the age group of 1 to 3 years, whereas GII.21 was reported in one child > 3 years of age. GII.13 was detected in two patients <1 year and > 3 years (Table 3).

### 3.5. NoV Genotype-Associated Clinical Outcomes

**Figure 4:**
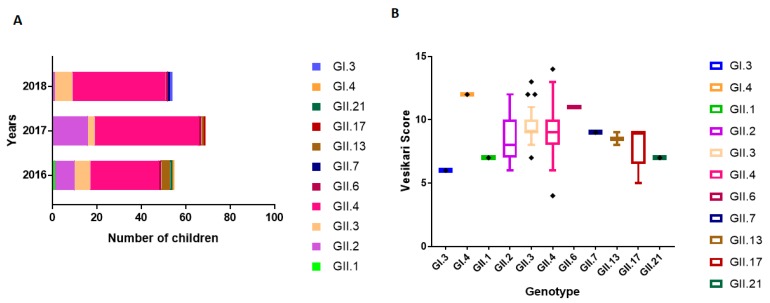
Prevalence and clinical outcomes of circulating NoV genotypes. (**A**) Percentage of circulating genotypes detected in Qatar, represented as years (Y-axis), and total number of positive cases (X-axis), respectively. (**B**) Association between genotypes and clinical illness in infected children as measured with Vesikari Clinical Severity Scoring System. The circulating genotypes are represented on the Y-axis, and the Vesikari score is represented on the X-axis (mild: score < 7, moderate: score 7 < score < 10), and severe: score:>10).

The prevalence of various NoV genotypes in children suffering from AGE between June 2016 and June 2018 in Qatar is represented in Figure 4A. Genotypes were also detected among hospitalized children during the study period: GII.1 (1/65, 1.5%), GII.6 (3/65, 4.6%), GII.7 (1/65, 1.5%), GII.13 (2/65, 3%), GII.17 (5/65, 7.7%), and GII.21 (1/65, 1.5%). We evaluated the association of clinical outcomes in hospitalized patients with circulating genotypes detected in Qatar using the Vesikari clinical severity scoring system (Figure 4B). Among genogroup GI, patient infected with GI.4 exhibited severe symptom with the highest Vesikari score (score ≥ 10), compared to the patient genotyped with GI.3 (score < 7). Among GII genotypes, we recorded higher severity score in patients infected with an uncommon genotype NoV GII.6 (score ≥ 10). Moderate disease severity (score: 7 < score < 10) was also observed among children infected with genotypes GII.4, GII.3, GII.2, GII.13, GII.17, and GII.7 (Figure 4B). On the other hand, disease severity (score ≥ 10) was higher among children infected with GII.4 viral strains closely related to recombinant GII.P16-GII.4/RUS/Novosibirsk/ 2017 (MG892929), GII.P4 New Orleans/2009/GII.4 Sydney/2012 (MG585810.1), GII.P16-GII.4 Sydney/2012 (KY887601), and GII.P16-GII.2 CHN/2017 (MH321823).

### 3.6. Seasonal Distribution of NoV Genotypes

**Figure 5:**
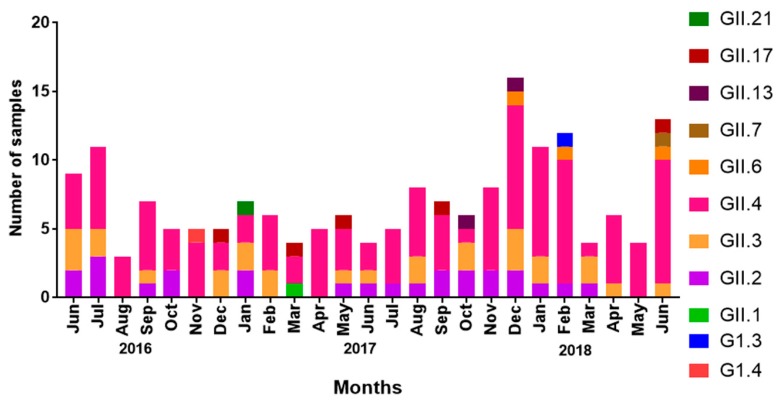
Seasonal distribution of NoV genotypes among children suffering from AGE. Y-axis represents the total number NoV-positive samples and x-axis represents circulating genotypes detected in affected children per month and year.

Overall, GII.4 was the dominant genotype throughout the study period and accounted for 62.2% (110/177) of all NoV cases. We observed that GII.4 was higher in summer (May–August) and lower in winter (October–December) in Qatar during 2016. However, in 2017, we observed sudden surge in GII.4 in winter, which lasted until February 2018. Notably, GII.2 was sporadically detected during the first year of the study (June 2016–June 2017), but then continuously detected during the second part of the study, almost in all months. On the contrary, GII.3 was detected throughout the year during the whole study period, with dominant peaks in June 2016 and December 2017 (Figure 5). It is worth noting that uncommon genotypes such as GII.6, GII.7, GII.17, GII.13, and GII.21 were observed at the end of summer (August–September) and winter (December–January) breaks in Qatar (Figure 5). Genogroups GI.3 and GI.4 were detected only once during the winter season (October–December) in 2016 and 2017.

### 3.7. NoV Genotypes Associated with the Nationality of Children

**Figure 6:**
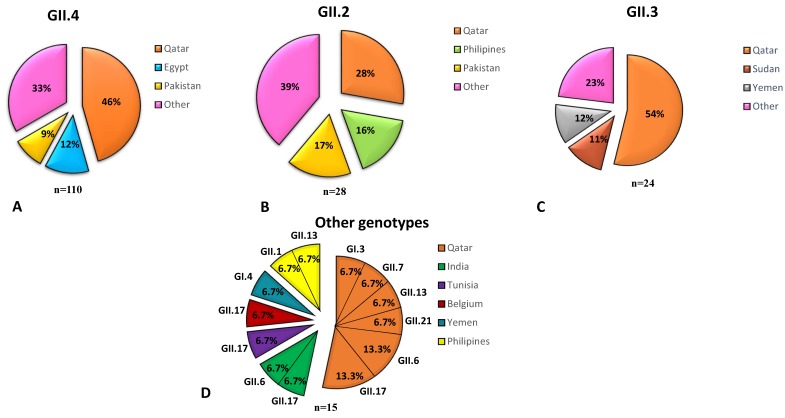
Pie chart representing the percentage of dominant NoV genotypes among top three nationalities. (**A**) Distribution of GII.4 (*n* = 110) among children from Qatar (orange color), Egypt (blue color), Pakistan (yellow color), and other nationalities (pink color). (**B**) GII.3 (*n* = 28) distribution among children from Qatar (orange color), Yemen (gray color), Sudan (brown color), and other nationalities (pink color). (**C**) GII.2 (*n* = 24) distribution among children from Qatar (orange color), Pakistan (yellow color), Philippine (light green color), and other nationalities (pink color). (**D**) Distribution of less prevalent genotypes (*n* = 15) in percentage with respect to their nationality. Qatar (orange color), Indian (green color), Tunisia (purple color), Belgium (marron color), Yemen (dark blue color), and Philippines (yellow color).

Qatar is a multinational country where expats represent more than 85% of its population. Therefore, we investigated the correlation of circulating genotypes and nationality of patients. All dominant genotypes in this study (GII.4 (62.2%), GII.2 (15.8%), and GII.3 (13.5%)) had higher incidence in Qatari children (Figure 6). The top three populations infected with GII.4 were Qatar (46%), Egypt (12%), and Pakistan (9%). GII.2 NoV infection was more circulating among children from Qatar (28%), Pakistan (17%), and The Philippines (16%). GII.3 was detected at higher rates among children from Qatar (54%), Yemen (12%), and Sudan (11%) (Figure 6). Uncommon GII genotypes were detected among both Qatari subjects (53.3%, 8/15) and expatriates (46.4%, 7/15) (Figure 6).

## 4. Discussion

In this current work, we report on the clinical, epidemiological, and molecular characterizations of NoV infections among children suffering with AGE in Qatar between June 2016 and June 2018. Several studies from the MENA region had evaluated the prevalence of NoV in pediatric population, including studies from Libya [35,36], Israel [37,38], Jordan [39], Yemen [39], Egypt [40], Tunisia [41,42], Iran [43], Morocco [44], Lebanon [45], and Turkey [44,46]. This study updates the knowledge obtained from other studies in the region; yet, it includes samples from mixed populations and correlates the findings to clinical manifestations.

Our analysis revealed the dominance of the GII genogroup, specifically the GII.4 (62.2%) genotype, which is consistent with previous reports from MENA region [23]. This observation is also in agreement with the global distribution of NoV, where GII.4 represents 68% of reported NoV cases worldwide [47].

In the MENA region, NoV is reported in both, winter and summer seasons, in countries such as Egypt, Morocco, Tunisia, and Turkey [23,48]. Similarly, NoV infections in Qatar were reported throughout the year, with a trend of lower infections in spring. We also assessed the variability of NoV infections in various age groups: Infants (< 1 year), toddlers (1–3 years), and young children (4–15 years). We reported higher NoV infections among children between 1–3 years of age compared to the other age groups. These observations are in contrast to previous report from Libya [35] where higher NoV rates were identified among children <1 year old. However, our report is in agreement with a previous study done by Oldak et al. (2012) in Northeastern Poland, in which higher NoV infection was detected in children between 1 and3 years of age [49]. The higher NoV infection rate in children between 1 and 3 years of age could be attributed to commingling of children in daycares, where children at this age have minimal immunity to the virus [50].

Nucleotide sequencing and phylogenetic analysis of partial polymerase and capsid proteins revealed that GII.4 (62.2%), GII.2 (15.8%), and GII.3 (13.6%) were predominant in our study. Circulating GII.4 strains shared homology with emergent recombinant strain GII.P16-GII.4/RUS/ 2017 (MG892929), GII.P16-GII.4 Sydney/2012 (KY887601) and GII.P4 New Orleans/2009/GII.4/Sydney 2012 (MG585810.1) (52). The Novosibirsk-2017 strain harbors several AA changes within the main epitope of the P2 capsid domain and shares a common ancestor with the NoV GII.4 variants Apeldoorn 2008 and New Orleans 2009 [51]. On the other hand, the Sydney-2012 strain was also isolated in the United States, Denmark, Japan, and Scotland [52,53,54,55]. The emergence of new NoV strains could be possibly due to the antigenic adaptation, which exerts a positive pressure as other studies, had suggested [53]. Such NoV GII.4 variants are known for their sudden emergence and quick outbreaks in communities, and have been associated with 65–80% of cases of NoV AGE worldwide [56].

GII.2 genotype was identified as the second most predominant cause of NoV infections in children in our study (16%). This genotype was detected at much lower rate in a recent study from Lebanon [45], where the it represented only 1.3% (1/76) of all NoV cases. Further, this genotype has been recently reported in Iran also at low frequency (2.8%), but not elsewhere in MENA region [45]. Most of GII.2 sequences in our study were closely related to the emerging strains GII.P16–GII.2 CHN/2017 (MH321823) from China. Moreover, GII.P16-GII.2 was reported to be the most predominant strain with a sudden increase in cases in Germany, France, and China in the period between 2016 and 2017 (winter) [57,58,59]. In China, this strain was the cause of 79% (44/56) of the outbreaks in 2016 [60]. Full-length analysis of GII.2 VP1 and RdRp region indicated that amino acid (AA) substitutions in partial RdRp in the GII.P16 are responsible for enhanced polymerase kinetics and thus, have been shown to be circulating at high frequencies predominating during the 2016–2017 season [61].

GII.3 was reported to be the most predominant genotype in Jordan in year(s) 2006 and 2007, contrasting reports from other countries around the globe [39]. This genotype ranked third in our study, with a similar trend of its prevalence in the study from Lebanon in 2016 [45]. The identified sequences showed closeness to the viral strains from Russia/2012 (KY596003.1), Brazil/2008 (KY702113.1), United Kingdom/2016 (KY887606.1), Johannesburg/ 2010 (KR904306.1), and Spain/2012 (KJ504419.1).

Other non-GII.4 genotypes were also detected in our study, including GII.1 (1/65, 1.5%), GII.6 (3/65, 4.6%), GII.7 (1/65, 1.5%), GII.13 (2/65, 3%), GII.17 (5/65, 7.7%), and GII.21 (1/65, 1.5%). These non-GII.4 genotypes were previously reported in the MENA region in Lebanon [45], Tunisia [62], Libya [35], Egypt [40], and Turkey [63]. Further genotypes GI.1, and GI.3, GII.1, GII.6, GII.7, GII.13, GII.17, and GII.21 were detected only once throughout the study. The low frequency of these genotypes did not allow any analysis to study their trend in the population.

A recent systematic review had shed light on the seasonality of NoV genotype circulation in the MENA region [23]. Among GI genotypes, GI.3 and GI.4 predominated, albeit that their prevalence was less than GII genotypes and were mainly observed circulating in winter season. Amid genogroup II, new GII.4 variants have been reported to emerge with unusual seasonality (spring/summer) [64], along with an increase during the winter season (average temperature: 22 ℃ and average humidity: 66%). In the MENA region specifically, dominance of GII.4 was reported in summer seasons in Morocco [44] and Libya [35]; and in winter season in Egypt (2006–2007) [65] and Tunisia (2011–2012) [62]. Interestingly, all of these four countries are North African countries that share similar climates. Moreover, the weather in Qatar is broadly grouped in two seasons: hot (May to October) and cool (December to February). March, April, and November are considered as warm transitional months [66]. Although GII.4 was predominant throughout our study period, its prevalence varied between seasons, with noticeable increase in the winter season of 2018. GII.2 was sporadically detected in the first year of our study, but then in almost all months starting from May 2017. No reports have been published about this genotype elsewhere from MENA region. On the other hand, GII.3 was predominantly reported during the winter season in Jordan (2014–2015) [39] and Tunisia (2011–2012) [62,67], but not in our study. It is worth noting that uncommon strains such as GII.6, GII.17, GII.13, and GII.21 emerged during the end of summer breaks in Qatar. This coincides with the return period of expats traveling to their native countries and Qatari nationalities prefer traveling to tourist places, explaining the introduction of different and new viral strains to Qatar yearly. It is not well understood why these strains do not widely circulate in the community, which requires further investigation.

We also analyzed NoV genotype correlation with the age of the infected children. The two GI strains were detected in children younger than three years of age. Regarding genogroup GII, GII.4 was detected at marginally higher rates among children aged 1 to 3 years reaching 55% (60/100) of the cases. GII.3 was clearly higher in toddlers (1–3 years of age), accounting for 67.5% (19/28) of reported cases. Contrarily, GII.2 was detected at higher rates in children <1 year of age compared to other groups, reaching 75% (18/24). All other uncommon GII genotypes were sporadically detected in all age groups.

Importantly, we investigated the correlation between circulating genotypes and clinical outcomes using the Vesikari Clinical Severity Scoring System. Such clinical association was previously reported in a NoV outbreaks in Taiwan (during 2006 and 2007, and 2011 and 2012), but not in the MENA region countries. In Taiwan, severe clinical symptoms were associated with different GII.4 variants (GII.4 2012a and GII.4 2012b) [68]. Hospitalized children infected with these genotypes presented a significantly higher incidence of intestinal hemorrhage, occult blood in feces, as well as high fever >39 °C compared to earlier outbreaks with GII.4 Sydney 2012 and GII.4 2006b [68]. Among genogroup GI, the severity score was higher among the patient infected with GI.4 (score ≥ 10), compared to patient infected with GI.3 (score < 7). Moderate disease severity (score: 7 < score < 10) was also recorded among children infected with genotypes GII.4, GII.3, GII.2, GII.13, GII.17, and GII.7. Increased frequency of diarrhea and vomiting (average frequency: 4–5 times/day) were reported in children infected with certain GII.4 strains, specifically those similar to GII.P16-GII.4/RUS/2017 (MG892929), GII.4 Sydney/2012, GII.P4 New Orleans/2009/GII.4 Sydney 2012 (MG585810.1), GII.P16-GII.4 Sydney/2012 (KY887601), and GII.P16-GII.2 CHN/2017 (MH321823) (score ≥ 10). On the other hand, infection with NoV GII.3 in children <1 year of age resulted severe clinical illness (score ≥ 10) compared to other age groups/other genotypes. In fact, only limited number of reports had correlated the serenity of NoV infection with the genotype among children. Few studies suggested that NoV GII-4 genotype has a greater virulence compared to other NoV genotypes [69,70].

We also investigated whether the circulating NoV genotypes were correlated to the country of origin of the subjects. The current work showed that the prevalence of NoV infection with GII.4 (43%), GII.3 (28%), and GII.2 (14%) were higher among Qatari children compared to other nationalities. Concerning uncommon genotypes, they were detected among both Qatari subjects (53.3%, 8/15) and expatriates (46.4%, 7/15).

Although GII.4, GII.2, and GII.3 genotypes have a significant role in NoV infections, reports have identified the epidemiological differences between these genotypes based on genetic polymorphism [20,71]. It has been suggested that the predominance of NoVs GII.4 may be due to higher evolution rate that enabled emergence of strains with better affinity binding to HBGAs [72,73]. In contrast, it was suggested that NoVs GII.2 and GII.3 had an evolutionary hindrance compared to NoVs GII.4 due to less processive polymerase and lower rate of evolution [20].

In conclusion, the results from this study are in line with global reports regarding the dominance of NoV GII, representing 98.8% of NoV infections [74]. Continuous surveillance for the circulating genotypes and their epidemiological and clinical significance is necessary, especially the country is preparing for World Cup 2022. Our study focused on a pediatric population and further studies including other population groups are required. Moreover, foodborne illness caused by NoV infection has been documented in Qatar previously [75] which stipulates the need of similar studies on food products, as more than 80% of vegetables/fruits being imported [76]. Furthermore, investigations focusing on whole genome sequencing of the circulating variants will give better insight about their evolution in the community, and thus will shed light on their potential source and how to eradicate them.

## Figures and Tables

**Table 1:** Demographic characteristics of study subjects.

Pediatric Profile	NoV-Positive Cases No. (%)	NoV-Negative Cases No. (%)	*p*-Value
**No. of Pediatric Patients**	177 (29.5)	423 (70.5)	-
Gender			
Female	77 (43.5)	235 (55.5)	0.0070
Male	100 (56.5)	188 (44.5)	
Age (years)			
<1 year	73 (41.2)	97 (23)	<0.0001
1–3 years	88 (49.7)	209 (49.4)	
>3 years	16 (9.1)	117 (27.6)	

**Table 2:** Clinical observations of NoV-positive cases *n* = 177 (29.5 %).

	NoV-Positive No. (%)	GI No. (%)	GII No. (%)
**Fever**			
Yes	70 (39.5)	0 (0)	70 (40)
No	107(60.5)	2(100)	105(60)
Max reached fever (°C)	40.6	38.6	39.9
Vesikari Score	7 ≤ scores ≤ 10	score < 7	score > 10
**Vomiting**			
Yes	165 (93.2)	2(100.0)	163(93.1)
Frequency (per day)	2–11	2–6	3–11
Duration (days)	1–7	3–4	1–7
No	12(6.8)	0 (0.0)	12 (6.9)
Vesikari Score *	score > 10	score < 7	score > 10
**Diarrhea**			
Yes	176 (99.4)	2(100.0)	174 (99.4)
Frequency (per day)	2–10	2–5	3–10
Duration (days)	1–5	3–4	2–5
No Diarrhea	1 (0.6)	0 (0.0)	1 (0.6)
Vesikari Score *	score > 10	(score < 7)	(score > 10)
**Dehydration**			
Severe	8 (4.5)	0 (0.0)	8 (4.6)
Moderate	76 (43)	0 (0.0)	76(43.4)
Mild	89 (50.2)	2(100.0)	87 (49.7)
No dehydration	4(2.3)	0 (0.0)	4 (2.3)
Vesikari Score *	7 ≤ scores ≤ 10	(score < 7)	(score > 10)
Mean Duration of Hospitalization (days)	1–7	2–3	1–7
Treatment **	24 (13.6)	0 (0.0)	24 (13.6)
Fully recovered	22 (91.7)	0 (0.0)	22 (91.7)
Partially recovered	2 (8.3)	0 (0.0)	2 (8.3)
Worsening	0 (0.0)	0 (0.0)	0 (0.0)
Recovered but became sick again	0 (0.0)	0 (0.0)	0 (0.0)
No treatment	153 (86.4)	2(100.0)	151 (85.3)
Fully recovered	129 (84.3)	2(100.0)	127 (82.3)
Partial recovered	24 (15.7)	0 (0.0)	24 (15.7)
Worsening	0 (0.0)	0 (0.0)	0 (0.0)
Recovered but became sick again	0 (0.0)	0 (0.0)	0 (0.0)

* Vesikari Score: mild symptoms (score < 7), moderate (7 ≤ scores ≤ 10), and severe (score > 10). ** Treatment: Ceftriaxone, Paracetamol, Cefexime, Electrolyte replacement solution, IVF, Salbutamol, Domperidone, NaCl, and Ibuprofen.

**Table 3:** Age-stratified NoV genotype detected in children with acute gastroenteritis (AGE) in Qatar.

Genotype	Total Number	<1 Year	1–3 Years	<1 Year
**Genotype I**				
GI.4	1 (0.56%)	1	0	0
GI.3	1 (0.56%)	0	1	0
**Genotype II**				
GII.4	110 (62.2%)	45	60	5
All non-GII.4 GII	65 (36.7%)	27	27	11
GII.1	1 (0.56%)	1	0	0
GII.3	28 (15.8%)	5	19	4
GII.2	24 (13.5%)	18	4	2
GII.6	3 (1.69%)	1	1	1
GII.7	1 (0.56%)	0	1	0
GII.13	2 (1.1%)	1	0	1
GII.17	5 (2.8%)	1	2	2
GII.21	1 (0.56%)	1	0	0

## Supplementary Materials

The following are available online at https://www.mdpi.com/1999-4915/11/5/400/s1, Figure S1: Phylogeny analysis of NoV circulating strains in children in Qatar.
