# Supplementary material for: Epidemiological, Molecular, and Clinical Features of Norovirus Infections among Pediatric Patients in Qatar
Source: Viruses. 2019 Apr 29;11(5):400. doi: 10.3390/v11050400 (PMC6563317; doi:10.3390/v11050400)
Supplement: Supplementary file 1 [file viruses-11-00400-s001.pdf]

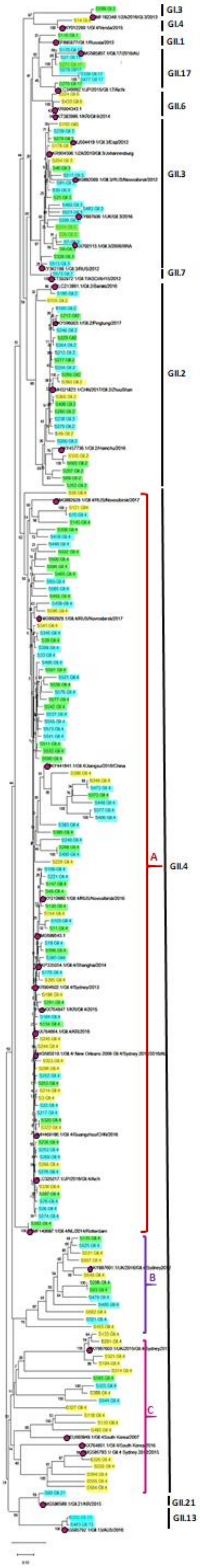

Supplementary Figure 1. Phylogeny analysis of NoV circulating strains in children in Qatar. The evolutionary tree was constructed for the junction between RNA-dependent RNA Polymerase (RdRp) and viral protein 1(VP1) using Neighbor-Joining method with 1000 bootstrap with MEGA7 [32]. The evolutionary distances were computed using the Poisson correction method and are in the units of the number of amino acid substitutions per site. NoV reference strains for each identified genotype in the present study are presented in purple dots. Disease severity of NoV AGE infected children by applying the Vesikari score system (30) according to clinical manifestations is highlighted for each sample: total score < 7 considered mild (green color); 7- ≦ 10 considered moderate (blue color); and >10 (up to 20) considered severe (yellow color).
